# Multi-strain volatile profiling of pathogenic and commensal cutaneous bacteria

**DOI:** 10.1038/s41598-020-74909-w

**Published:** 2020-10-21

**Authors:** Shane Fitzgerald, Emer Duffy, Linda Holland, Aoife Morrin

**Affiliations:** 1 School of Chemical Sciences, National Centre for Sensor Research, Insight SFI Research Centre for Data Analytics, Dublin City University, Dublin, Ireland; 2 School of Biotechnology, Dublin City University, Dublin, Ireland

**Keywords:** Bacteria, Bioanalytical chemistry, Mass spectrometry

## Abstract

The detection of volatile organic compounds (VOC) emitted by pathogenic bacteria has been proposed as a potential non-invasive approach for characterising various infectious diseases as well as wound infections. Studying microbial VOC profiles in vitro allows the mechanisms governing VOC production and the cellular origin of VOCs to be deduced. However, inter-study comparisons of microbial VOC data remains a challenge due to the variation in instrumental and growth parameters across studies. In this work, multiple strains of pathogenic and commensal cutaneous bacteria were analysed using headspace solid phase micro-extraction coupled with gas chromatography–mass spectrometry. A kinetic study was also carried out to assess the relationship between bacterial VOC profiles and the growth phase of cells. Comprehensive bacterial VOC profiles were successfully discriminated at the species-level, while strain-level variation was only observed in specific species and to a small degree. Temporal emission kinetics showed that the emission of particular compound groups were proportional to the respective growth phase for individual *S. aureus* and *P. aeruginosa* samples. Standardised experimental workflows are needed to improve comparability across studies and ultimately elevate the field of microbial VOC profiling. Our results build on and support previous literature and demonstrate that comprehensive discriminative results can be achieved using simple experimental and data analysis workflows.

## Introduction

The production of VOCs by microorganisms in different media and biological fluids has been investigated for many years with the aim of characterising various disease-specific odours^1^. VOC profiling studies of pure bacterial cultures are needed to identify the cellular origin of metabolites associated with specific phenotypes of pathogens under specific conditions^2^. Untargeted profiling analyses investigating respiratory infections in patients with cystic fibrosis^3,4^, pneumonia^5^, and tuberculosis^6^ have demonstrated the discriminatory power and diagnostic potential of VOCs. These analyses rely on the identification of trends in VOC profiles between various disease-associated subjects and healthy subjects. The ‘top down’ workflow generally consists of analysing whole VOC profiles via multi-variate analysis techniques such as principal component analysis (PCA) to discriminate between the two groups^5,6^. The same approaches are typically employed in bacterial VOC profiling studies to investigate species-level diversity. Studying VOCs emitted from bacteria under controlled conditions identifies potential mechanisms behind projected VOC profiles in infectious disease-associated individuals.

Bacteria produce VOCs as side-products of primary metabolism and secondary metabolism^7^. The aim of primary metabolism is to simply metabolise all available glucose and derive as much adenosine triphosphate (ATP) as possible, which occurs during the exponential growth phase^8^. Secondary metabolism occurs in the stationary growth phase—under resource-limited conditions—and involves the further metabolism of primary metabolites^7^ and fermentation processes that generate alcohols and acetate^8^. The biosynthesis and subsequent metabolism of fatty acids (FAs) are both multi step processes that can generate VOCs at each individual step^8,9^. FAs are produced from acetyl CoA (or propionyl-, isobutyryl-, isovaleryl-, or 2-methylbutyryl-CoA), which are extended with malonate units to assemble various fatty acids. They are then metabolised by the β-oxidation pathway^10^. The processes consist of multiple decarboxylation, hydrolysis, and reductions which generate a variety of alkanes^11^, 1-alkenes^11,12^, methyl ketones^13,14^, and 1-alkanols^9,15^. Microbes can also metabolise amino acids to produce volatile short-chain FAs^9,16,17^ such as 3-methylbutyric acid^18^. Proposed microbial metabolic pathways of VOC production are discussed further elsewhere^9^.

Previous studies have shown that bacteria have species-specific VOC profiles that are directly influenced by growth parameters such as growth media^19,20^, incubation time^21,22^, oxygen content (headspace volume)^23^, temperature^24^ and pH^25^. The results from studies are also influenced by the sampling techniques employed. Frequently used sampling techniques coupled with GC–MS include SPME^26,27^, thermal desorption tubes^21^, direct syringes^28,29^. Direct detection methods such as SIFT-MS^26^, SESI-MS^30^ and PTR-MS^31^ have been previously employed for real-time analysis of VOCs, however, the resulting VOC profiles obtained from these methods typically contain low numbers of compounds. The variation in growth parameters and instrumental techniques across studies make inter-study comparisons difficult and highlights the need for more supporting literature and comprehensive data. The mVOC database^32^ (updated to mVOC 2.0^33^) contains thousands of logged VOCs from a wide range of microbes, as well as proposed metabolomic pathways. Databases such as the mVOC 2.0 database have the potential to emerge as invaluable tools in the field of VOC profiling, allowing rapid cross-study validation of results. Building comprehensive databases, in turn, will require a wide collaborative effort and researchers should be encouraged to upload their results to these developing databases.

In this work, we used a rapid HS-SPME-GCMS workflow to obtain the VOC profiles of multiple strains of prevalent wound pathogens^34,35^ *S. aureus, P. aeruginosa,* and *E. coli*, as well as multiple strains of the skin commensal *S. epidermidis* and media controls. It is estimated that around one in four people with diabetes will develop a diabetic foot ulcer (DFU) in their lifetime^36^. Infections of DFUs are directly associated with poor outcomes^35^ and ulcer duration is closely associated with species-level diversity^37,38^. It has also recently been demonstrated that strain-level diversity in wound infections is also associated with infection severity^38^. Rapid non-invasive discrimination of bacteria at the species- and strain-level could therefore potentially speed up the turnover of clinical information and greatly contribute to the clinical workflow. However, while is a long-term objective of our group and currently, understanding the mechanisms governing species- and strain-specific VOC profiles is our primary objective. We primarily aimed to investigate species-level variation in the VOC profiles of these bacteria using multi-variate analysis techniques. Secondary to this, we wanted to assess the feasibility of VOC profiling for measuring strain-level differences between two strains of each species of these bacteria. The final aim was to monitor VOC emissions of individual strains of *S. aureus* and *P. aeruginosa* over 48 h to assess the relationship between the kinetics of bacterial VOC profiles and their respective growth phases. Untargeted bacterial VOC profiling has the potential to be employed as a non-invasive characterisation tool for a range of infections including cutaneous disorders and chronic wounds. This work serves to identify species- and strain- specific cellular metabolites and metabolomic trends that could potentially support and aid interpretation of observed trends in future untargeted studies.

## Results

### Comparative analysis of volatiles emitted from planktonic bacteria cultures

**Figure 1:**
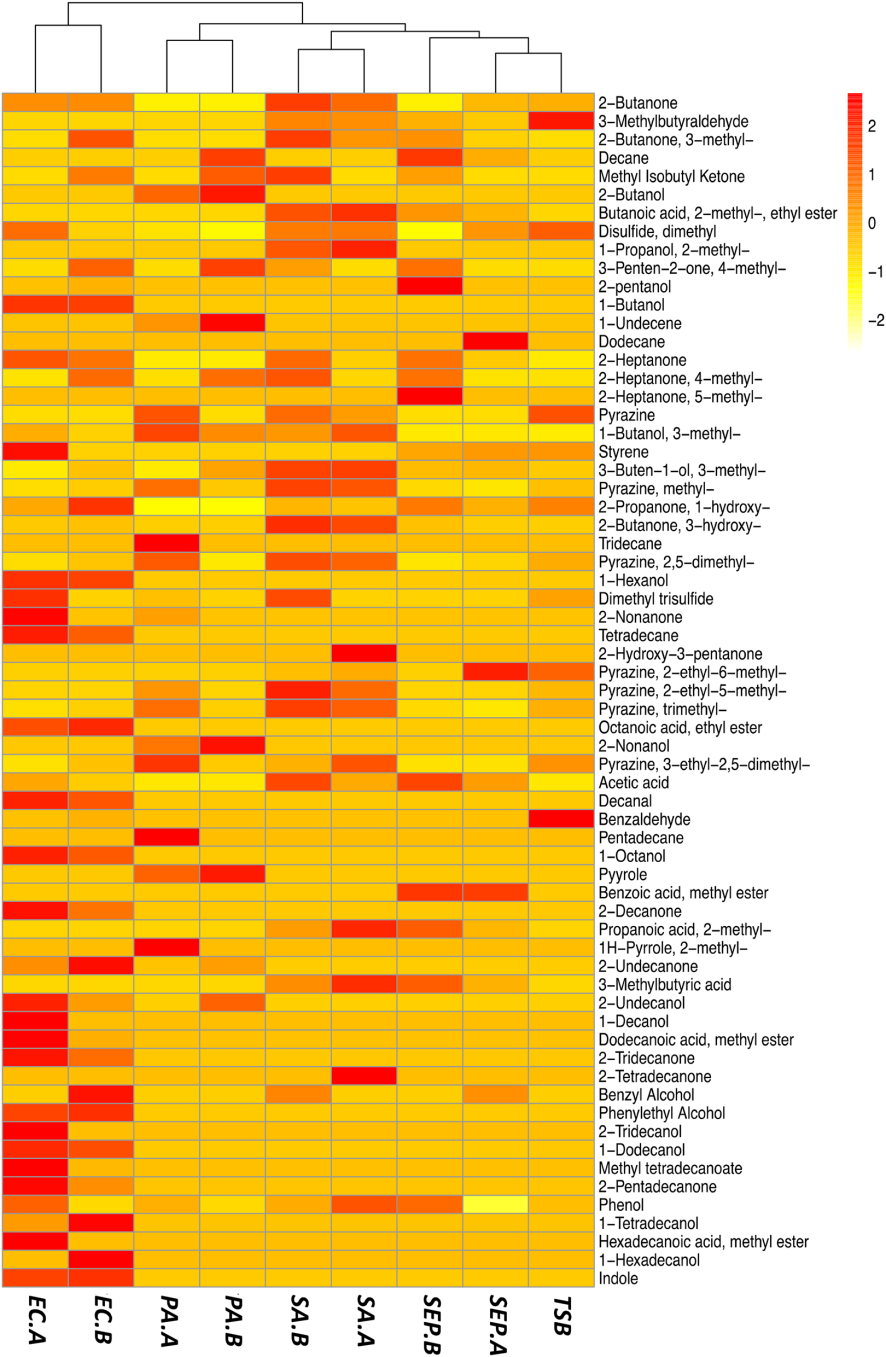
Heatmap showing the mean abundance of VOCs recovered (rows) from each bacterial strain (columns). Values were scaled and centred by their respective rows, with highly abundant VOCs being coloured red, and less abundant VOCs being marked orange–yellow.

GC–MS analysis of the VOCs recovered from the SPME fibers showed that there was a wide variety of compound classes present in the HS of the bacterial cultures. Numerous blank samples were collected and analysed to identify and exclude exogenous compounds from the SPME fiber, glass vial, and column. A total of 65 compounds were identified from the bacterial and control samples (see Fig. 1). Of these, 19 compounds were found in the HS of media control samples. Following 24 h incubation, *S. aureus, P. aeruginosa, E. coli*, and *S. epidermidis* all generated characteristic VOCs. Two individual strains of each species of bacteria were cultured and analysed in triplicate. Compound identification was performed and RI matched VOC profiles were established for each bacterial strain and integrated into a complete dataset, which incorporated all species, strains and controls tested and the compounds identified. An initial visual inter-strain comparison was performed by overlaying the chromatograms (Figures S1–S4) which demonstrate a high degree of similarity between the VOC profiles of respective strains.

Hierarchical clustering was performed to visualise the similarities across the VOC data. It is a statistical method used to classify multiple objects into groups (clusters) based on similarities between them. The results are visualised as a dendrogram (Fig. 1). Dendrograms are bottom-up representations of the clustering procedure; each object is initially assigned to its own cluster, and these individual clusters are grouped together based on their similarity. The clustering algorithm then progressively joins similar clusters together until all objects are grouped by a single cluster. The length of an edge between a cluster and its split is proportional to the dissimilarity between the split clusters^39^. Figure 1 visualises the clustering results coupled with a heatmap to show the different patterns in individual VOC abundances across all bacterial strains. The heatmap contains 65 rows, which are labelled by each VOC, and listed in order of increasing retention time. Each column of the heatmap represents the mean VOC abundance recovered from each strain (n = 3). Figure S5 in the SI visualises the inter-sample variation across each bacterial sample of each examined species and strain. In Figure S5, compounds found in the TSB growth media. In Fig. 1, the relative VOC abundances are visualised using a gradient red/yellow colour scale, where dark orange/red represents a high abundance; and orange/yellow represents a low abundance. All bacterial strains—except for *SEP.A*—were successfully clustered with their respective species. The Euclidean distance between the *E*. *coli* strains and the other bacteria tested was the greatest, verifying that the two *E. coli* strains had the most discriminative VOC signatures of all samples tested. S. *epidermidis* strains emitted a lower number of VOCs in comparison to the other species and were clustered close to the media control as a result. *S. aureus* and *P. aeruginosa* both have clearly differentiated VOC signatures, which can be seen from the heatmap by the relatively high number of unique ‘red’ values.

**Figure 2:**
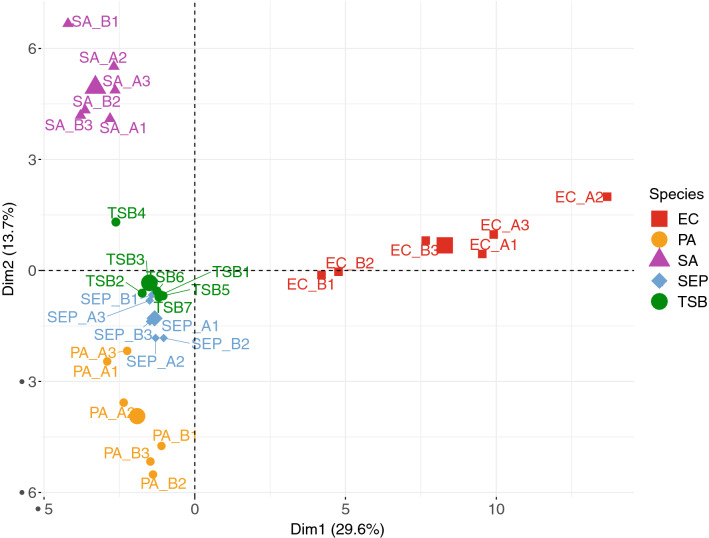
Scores plot representation of scores of bacterial samples. PC1 and PC2 summarised 43.4% of the variance of the overall dataset, with 29.6% being summarised by PC1 and 13.7% being summarised by PC2.

Principal component analysis (PCA) was performed to summarise the dissimilarities in the data. PCA reduces the dimensionality of the data by identifying characteristic VOCs, and using them to construct new linear variables called principal components (PCs), along which the variation is maximal. The PCs are variables consisting of linear combinations of the original variables; which can then be visualised using scores plots. Scores plots show inter-unit distances and visualise species- and sample-like patterns revealed by the PCA to identify groups that characterise the overall dataset^40^. The PCA results shown in Fig. 2 show that the bacterial samples have clustered successfully to their respective species. The highest inter-sample diversity was observed in *E. coli* samples where the inter-strain and -sample variation is summarised by PC1*. S. aureus, P. aeruginosa* and *S. epidermidis* discrimination—relative to each other—is summarised by PC2, whereas their discrimination from *E. coli* is summarised on the x-axis by PC1. The loadings plot shown in Figure S6 visualises the individual contribution of each bacteria-specific VOC to the overall differentiation of the examined species. Compounds found in the TSB media were subtracted from Figure S6 to improve differentiation of bacterial samples.

**Figure 3:**
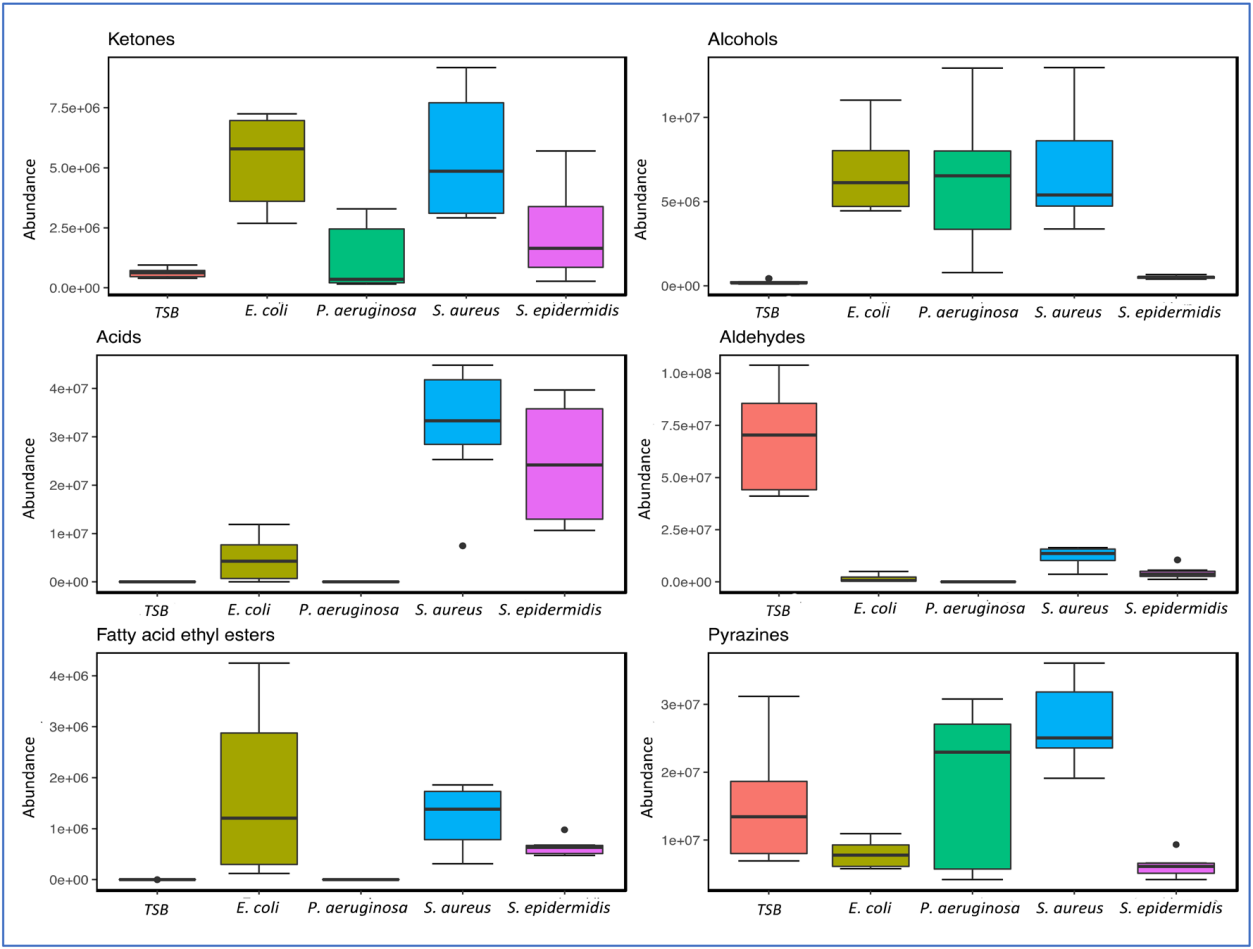
Grouped boxplots showing the abundance of ketones (top left), alcohols (top right), acids (middle left), aldehydes (middle right), fatty acid ethyl esters (bottom left), and pyrazines (bottom right) recovered from the control and each species of bacteria (t = 24 h). Respective strains were grouped together to clearly summarise the species-level discrimination across the data. The boxes represent the interquartile range: the line running across each box represents the 50th percentile (median), the top of the box represents the 75th percentile, and the bottom of the box represents the 25th percentile. The whiskers (error bars) represent either the smallest or largest value within 1.5 times the interquartile range above the 75th percentile or below the 25th percentile. The black dots above or below the boxes represent outliers that are greater than 1.5 times the interquartile range beyond either end of the box.

The distribution of the major compound classes recovered from all tested species of bacteria and controls is summarised in Fig. 3. Ketones were recovered from all bacterial samples (Fig. 3) and the controls. The largest number of ketones were recovered from *E. coli* samples. The relative abundance of individual ketones can be seen in Fig. 1. Long chain methyl ketones such as 2-undecanone, 2-tridecanone, and 2-pentadecanone were recovered from *E. coli*. High abundances of 3-hydroxy-2-butanone (acetoin) were observed in all *S. aureus* chromatograms. Acetoin was also detected in *S. epidermidis* and *E. coli* samples, but in lower amounts compared to that of *S. aureus*. *P. aeruginosa* was found to emit a lower number of ketones than other species tested. Both *P. aeruginosa* strains were found to emit low abundances of 2-undecanone, while 2-nonanone was only detected in *PA.B* (Fig. 1)*.* 2-Butanone, 3-methyl-2-butanone, and 1-hydroxy-2-propanone were all detected in the media controls.

Alcohols were recovered in high abundances from all bacterial samples and low abundances from the controls. In Fig. 3, the median abundance value for alcohols was similar for *E. coli, S. aureus,* and *P. aeruginosa* samples; the abundance of alcohols in *S. epidermidis* and control samples was relatively lower. Out of the bacteria tested, some alcohols were shared between species, and others were unique to individual species. 3-Methyl-1-butanol (isoamyl alcohol) was detected in every strain of bacteria and was particularly abundant in all *S. aureus* and *PA.A* samples (Fig. 1). 3-Methyl-3-buten-1-ol was detected in each strain except for *PA.B* and *EC.A*. 2-Butanol and 2-nonanol were extracted from both *P. aeruginosa* strains, though 2-undecanol was only extracted from the *PA.B* strain. We identified various 1- and 2-alcohols from both *E. coli* strains, these included 1-hexanol, 1-octanol, 1-decanol (only *EC.A)*, 2-undecanol, 2-tridecanol (*EC.A)*, 1-tetradecanol, and 1-hexadecanol (only *EC.B*). Low abundances of 1-dodecanol and benzyl alcohol were the only alcohols detected in the blank growth media.

High abundances of acids were detected in all *S. aureus* and *S. epidermidis* samples, and to a lesser extent in the *E. coli* samples; while none were detected in the control or a in *P. aeruginosa* samples (Fig. 3)*.* High abundances of 3-methylbutyric acid and acetic acid; and relatively lower abundances of propanoic acid, 2-methyl- and were observed in all *S. aureus* and *S. epidermidis* chromatograms. High abundances of acetic acid were also detected in *E. coli* samples (Fig. 1). No acids were detected in the control samples.

Aldehydes were detected in low abundances in *S. aureus, E. coli,* and *S. epidermidis* samples, and in high abundances in the controls (Fig. 3). 3-Methylbutyraldehyde was detected in moderate abundances in *S. aureus* samples and lower abundances in *S. epidermidis* samples (Fig. 1). Low abundances of decanal and benzaldehyde were detected in *E. coli* samples (Fig. 1). No aldehydes were recovered from any *P. aeruginosa* samples. A high abundance of aldehydes such as 3-methylbutyraldehyde and benzaldehyde were detected in the control samples (Fig. 3).

Fatty acid ethyl esters were detected in *E. coli, S. aureus* and *S. epidermidis* samples. The highest number of individual fatty acid ethyl esters were detected in *E. coli* samples (Fig. 1), whereas *S. aureus* samples had the highest median abundance of fatty acid ethyl esters (Fig. 3). Butanoic acid, 2-methyl, ethyl ester was detected in *S. aureus* (relatively high abundance) and *S. epidermidis* (relatively mid abundance); long-chain compounds such as dodecanoic acid, methyl ester, methyl tetradecanoate, and hexadecenoic acid, methyl ester were detected in *E. coli* samples. No fatty acid ethyl esters were detected in the control samples.

Pyrazine compounds were detected in all bacteria samples. All pyrazines were also detected in all media controls. Variation seen in Fig. 3 could be a result of batch variation, as it can be seen that the error bars of the growth media control box covers the interquartile range of the other species tested.

There were multiple characteristic compounds detected that didn’t fall into the compound classes discussed above. 1-Undecene and pyrrole in *P. aeruginosa* samples (2-methyl-1H-pyrrole was only present in the HS of *PA.B)*. Indole (the most abundant compound detected out of all the bacterial samples) was detected in all *E. coli* samples. Styrene was detected in *E. coli* samples, *S. epidermidis* samples and in very low abundances in control samples.

### Kinetic study of *S. aureus* and *P. aeruginosa* VOC production

**Figure 4:**
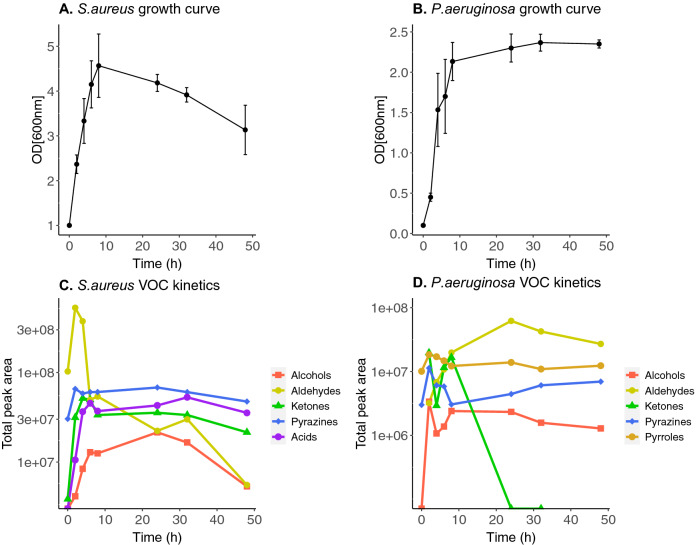
Bacterial growth curves of (**a**) S. aureus (n = 3) and (**b**) P. aeruginosa (n = 3) over an incubation period of 48 h OD_600_ measurements were taken at 2, 4, 6, 8, 24, 32, 48 h. Kinetic plots of (**c**) S. aureus and (**d**) P. aeruginosa showing the changes in VOC abundances of different compound classes over an incubation period of 48 h.

To investigate the relationship between VOC emission and bacterial growth stage, growth curves for individual *S. aureus (SA.A*) and *P. aeruginosa (PA.B*) strains were constructed (Fig. 4a,b) and compared with VOC emission abundances of specific compound classes over time (Fig. 4c,d). Growth curves were constructed from OD_600_ readings taken at defined time points over 48 h. Emission kinetics plots for individual compounds can be found for *S. aureus* (Figure S7) and *P. aeruginosa* (Figure S8) in the SI. VOCs were sampled using SPME at equivalent time points over this same period.

It was necessary to allow the HS of the samples to equilibrate at 37 °C prior to VOC sampling. Due to this, the 0 h data points used for VOC abundances (Fig. 4c,d) were taken from a set of blank media controls equilibrated at 37 °C.

The constructed growth curves allow the visualisation of the growth stage of an organism. In this case, OD_600_ was used as a measure of the turbidity of a bacterial liquid culture—as bacterial cells multiply, the liquid culture becomes more turbid—is used to assess bacterial growth stage^41^. Typically, organisms proceed along the path set by the bacterial growth curve, passing through four characteristic stages: lag, log (exponential growth), stationary, and decline. The lag phase is a distinct growth phase whereby the organisms are adapting to their new environment and preparing for rapid growth^42^. The first OD_600_ measurement for these experiments was taken after 2 h and indicated that the bacteria were already in the log phase of growth. This can be clearly seen in both *S. aureus* and *P. aeruginosa* samples as a rapid increase in the OD between 0 and 8 h (Fig. 4a,b). During this log phase all available resources such as glucose and fatty acids are consumed. When energy sources become limited, the bacteria then enter the stationary phase of growth, and activate reserve pathways that enable the metabolism of secondary substrates to survive^43^. *S. aureus* cell growth decreased between 8 and 24 h, therefore the stationary phase of growth is not visible in Fig. 4a; while *P. aeruginosa* remained in the stationary growth phase from 8 to 48 h (Fig. 4b).

Overall, Fig. 4 shows that the VOC abundances for certain compound classes change with respect to growth phase for both species. For example, aldehyde abundances decreased in *S. aureus* samples in the first 8 h (exponential growth phase) of incubation, and then continued to decrease over the following 40 h but at a slower rate (Fig. 4c). A similar trend was observed in *P. aeruginosa* with aldehyde abundances decreasing to minimal levels by approximately 24 h (Fig. 4d). The initial rapid decrease in aldehyde abundances likely indicates a rapid consumption of the aldehydic compounds present in the growth media during the exponential growth phase. In *S. aureus* samples, the exponential growth phase was also marked by proportional exponential increases in abundances of acids, ketones, and alcohols (Fig. 4c). In *P. aeruginosa* samples, an increase in alcohol abundance was observed in the exponential growth phase, as well as an emission of pyrrole after 2 h incubation (Fig. 4d). Abundances of pyrazines did not change significantly over 48 h for either species which indicated that no pyrazine compounds were consumed or produced by either bacteria over the course of the experiment.

The final phase of the bacterial growth curve is the death phase which is characterised by the net loss of bacterial cells, where the rate of cell death is greater than the rate of cell production due to unsuitable conditions such as exhausted nutrients and lack of oxygen^44^. The death phase of *S. aureus* cells occurred between 8 and 24 h and was marked by a depletion in aldehyde abundances. In *S. aureus* samples, between 8 and 24 h, alcohol abundance increased as the aldehyde abundance decreased, suggesting that aldehydes were metabolised into alcohols. From 24 to 32 h, a reduction in alcohol abundance simultaneously occurred with a comparable increase in acid abundance, which indicated that the alcohols were further metabolised into acids. Abundances of acids, alcohols, aldehydes, and ketones all subsequently declined from 32 to 48 h. In *P. aeruginosa* samples, the growth curve shows that *P. aeruginosa* cells remained in the stationary growth phase from approx. 8 to 48 h. During this period there was no significant change in emission of alcohols and ketones. The cumulative abundance of pyrrole compounds appeared to increase and decrease in the early growth phase between 2 and 4 h, which was followed by another increase from 4 to 8 h, before gradually declining in the later stages of the experiment (24–48 h).

## Discussion

In this study we used HS SPME coupled with GC–MS to collect and identify VOCs emitted from multiple strains of pathogenic and commensal species of bacteria. Using multi-variate analysis techniques such as hierarchical clustering (Fig. 1) and PCA (Fig. 2) to reveal patterns in the data, each species was successfully discriminated based on their respective VOC profile. All pyrazine compounds identified in this study were detected in the blank growth media samples as well as the bacterial samples. Although the generation of these particular compounds by bacteria has been described^8^, many have been previously linked to the thermal sterilisation process of growth media via autoclaving^9^. The skin commensal, *S. epidermidis*, emitted a relatively low number of VOCs. Acetic acid, 3-methylbutyric acid and 3-methylbutyraldehyde were among the VOCs recovered from its HS. These compounds were also highly abundant in all *S. aureus* samples. *Staphylococcal* species catabolise amino acids found in the growth media to 3-methylbutyraldehyde, which is then oxidised by an aldehyde dehydrogenase to form 3-methylbutyric acid^18^ (isovaleric acid). 3-Methylbutyric acid is a characteristic VOC emitted by various *Staphylococcal* species^45,46^ and is strongly associated with the generation of human body odour^16,47^. *S. aureus, S. epidermidis,* and *E. coli* also produced the high amounts of 3-hydroxy-2-butanone (acetoin). Acetoin is an uncharged molecule—produced by bacteria through the conversion of pyruvate and is known to prevent the over-acidification of the intracellular environment^48^. It can be seen in Fig. 1 that the *S. epidermidis* strains did generate characteristic VOC profiles, but they were clustered close to the media control due to the limited number of species-specific VOCs recovered from the HS of the samples. Coagulase-negative *Staphylococci* such as *S. epidermidis* have been previously reported to exhibit a relatively slow metabolism of carbohydrates when compared to pathogenic bacteria^49,50^. Despite this, in a recent comparative study, the VOC profiles of *S. epidermidis* were reported to be highly dependent on the growth media used^20^. *S. epidermidis* cultures emit a relatively lower number of VOCs when cultured in TSB media^20^, compared to other growth media; this would suggest that our choice of growth media was a potential factor that influenced the limited VOC profiles of *S. epidermidis* observed here.

*P. aeruginosa* emitted a stable set of compounds that allowed it to be clearly discriminated from the other species tested, whereby the notable VOCs found here have all been previously reported. The VOC profiles obtained from the *P. aeruginosa* strains are similar to those reported by Bean et al.^51^ and Filipiak et al.^21^. The most abundant compounds recovered from the *P. aeruginosa* HS were 3-methyl-1-butanol and 1-undecene. 3-Methyl-1-butanol was common to all bacteria tested while 1-undecene was unique to *P*. *aeruginosa* and has been previously reported to be produced through the fatty acid metabolism^9^. Acetoin has been previously reported to be emitted by *Pseudomonads*^19^, and was detected in relatively low abundances during the early growth phase of *P. aeruginosa* (Figure S7). Pyrrole is a unique nitrogen-containing compound that was detected and has also been previously reported^21,51^. 2-Nonanone (only detected in PA.A) and 2-undecanone were detected and have been previously reported as being potentially specific to *P. aeruginosa* biofilms^28^. However the results obtained from this study and other studies^21,51,52^ show that these specific compounds are also emitted by planktonic cultures. We did not detect 2-aminoacetophenone—an odorous VOC frequently reported in *P. aeruginosa* VOC profiles^53,54^—in any of the samples in either strain.

The *E. coli* strains emitted the largest number of VOCs and had the most distinctive VOC profiles of the four species tested (Figs. 1, 2). Acetic acid was present in all *E. coli* samples; which has been previously reported to be a product of anaerobic respiration of carbohydrates^55^. Compounds such as styrene and a variety of fatty acid ethyl esters were extracted from the HS of the *E. coli* samples. This is in agreement with the literature reports on the biosynthesis of these compounds by *E. coli*, which has recently gained interest in the biofuel industry due to the petrochemical properties of these compounds^56,57^. Indole was the most abundant compound recovered from the HS of *E. coli*. It is commonly found in human faeces as a product of *E. coli* activity in the human gut^58^, and its high abundance is likely responsible for the characteristic foul odour of the culture. The detection of 1-alcohols such as 1-butanol, 1-hexanol, 1-octanol, and 1-decanol was in agreement with existing literature^22,59,60^. Indole is the major VOC produced by *E. coli*^9^ and has been documented as an intercellular signal molecule amongst diverse bacteria^61^. It is produced by over eighty species of bacteria, though very few produce comparable abundances to *E. coli*^61^. From this, it may be possible to identify the presence of *E. coli* in a real sample from the abundance of indole recovered.

There was limited strain-level diversity observed in VOC profiles. Differences between the two *E. coli* strain profiles included the presence of 1-decanol and 2-tridecanol in *EC.A* samples only; and 1-hexadecanol being present only in *EC.B* samples (Fig. 1 and Figure S5). Variation between the two *E. coli* strains can be seen clearly in Figure S5 where the triplicate samples of EC.A form their own independent cluster, clearly discriminating it from the rest of the bacterial samples. Another example of strain-level variation was between the two *P. aeruginosa* strains, where 2-nonanone was only present in PA.A samples, and 2-undecanol was only present in PA.B samples. Strain-level diversity observed in *S. aureus* and *S. epidermidis* was primarily due to varying abundances of compounds emitted between strains (Fig. 1 and Figure S5). Quantitative strain-level discrimination of bacterial VOC profiles via SIFT-MS has been previously reported for *E. coli* and *Proteus Mirabilis*^62^*.* However, the number of compounds detected via SIFT-MS appears to be limited across studies^62,63^, and analyses of more comprehensive VOC profiles are required to confirm the prospect of strain-specificity. Bean et al. identified a total of 391 compounds across 24 clinical *P. aeruginosa* isolates taken from 8 different sites of the body^51^. They assessed strain-level diversity via hierarchical clustering of the VOC profiles and found that although 4 of isolates taken from the eye clustered together, there was not enough evidence across the rest of the data to suggest that *P. aeruginosa* strains can be differentiated. In our study, overall, there were no significant differences in the whole VOC profiles between strains (*E. coli*, p = 0.484; *S. aureus*, p = 0.472; *P. aeruginosa*, p = 0.434; *S. epidermidis*, p = 0.113). Although the potential practical application of differentiating bacteria at the strain-level has been shown through VOC profiling of bacteria with varying antibiotic-resistances and sensitivities^64^, further research is required in this area in order to deduce the specific factors affecting strain-level diversity of microbial VOC profiles.

Temporal VOC profiles of *S. aureus* and *P. aeruginosa* strains were analysed relative to the varying growth and development of cells. These pathogens were chosen for this analysis as they have different physiological classifications and are both highly prevalent in diabetic wound infections. The plots shown in Fig. 4 demonstrate the relationship between the emission of particular compound classes and the growth of cells. The plots shown in Figures S6 and S7 visualise the emission of individual compounds against time. It can be seen in both kinetic plots (Fig. 4c,d) that aldehyde abundances sharply decreased following the incubation of both *S. aureus* and *P. aeruginosa* samples. As aldehydes were predominantly found in the TSB growth media (Fig. 4c,d (t = 0 h)), it is highly likely that compounds such as benzaldehyde and 3-methylbutyraldehyde were rapidly metabolised by the bacteria and reduced to alcohols^18,65^. Aldehydes (e.g. 3-methylbutyralde) can be reduced to alcohols (e.g. 3-methyl-1-butanol) via alcohol dehydrogenases, or oxidized to acids (e.g. 3-methylbutyric acid) via aldehyde dehydrogenases^18^. *P. aeruginosa* has been reported to metabolise aldehydes very efficiently^18,21^. 3-Methylbutyraldehyde has been described as a marker of *S. aureus* growth—and is a known precursor of 3-methylbutyric acid^18^, however it was also found in the HS of blank media controls. We observed an eightfold increase in the abundance of 3-methylbutyraldehyde between 0 and 2 h which indicated that *S. aureus* was emitting this compound in the early phase of growth (Figure S6), which was followed by a steady decline of 3-methylbutyraldehyde from 2 to 48 h.

Secondary metabolism of alcohols, aldehydes, fatty acids, and ketones generate many volatile intermediary compounds via reversible reactions, and generate various lipids, alkanes and alkenes as irreversible end products of these pathways^9,65^. As the abundance of viable *S. aureus* cells decreases (Fig. 4a), there are indications of secondary metabolism in Fig. 4c at 8–24 h, where aldehyde abundances decrease further as the alcohol abundances increase, and then at 24–32 h where alcohol abundances decrease as the acid abundances increase. In this study, the decline of acids, aldehydes, alcohols and ketones from 32 to 48 h could suggest that these metabolic pathways have been exhausted and that these compounds have been gradually degraded into lipid or hydrocarbon end products^65^.

In Fig. 4d, it can be seen that as *P. aeruginosa* cell growth stagnates in the stationary phase between 24 and 48 h, the emission rate of alcohols, pyrrole, and ketones is arrested and there is no further net increase in any of these chemical classes. Volatile nitrogen-containing compounds such as pyrrole have been previously reported to reach a maximum abundance after a short period of incubation, and then gradually degrade over time^21^. In our study, the cumulative abundance of pyrrole compounds (2-methyl-1H-pyrrole and pyrrole) reached a maximum after 2 h incubation. 2-Methyl-1H-pyrrole was only detected at 2 and 8 h, while pyrrole was detected at every timepoint and reached maximum abundance at 24 h (Figure S7). Degradation mechanisms for pyrrole are not described in the literature. However, the cumulative abundance of pyrrole was essentially halved between 24 and 48 h, which is in agreement with the finding reported by Filipiak et al.^21^ The overall patterns observed in bacterial VOC profiles in response to the growth and death of cells suggests that the detection and monitoring of VOCs could potentially provide a non-invasive means of metabolically tracking bacterial growth.

## Conclusion

The aims of this study were to obtain the VOC profiles of multiple strains of four prevalent bacterial species present in infected wounds; to investigate species- and strain-level diversity in the VOC profiles obtained; and to assess how VOC profiles of *S. aureus* and *P. aeruginosa* were affected with respect to cell growth. Comprehensive VOC profiles for each examined strain were obtained using HS-SPME GC–MS. Examined strains emitted a variety of compound classes that allowed clear species-level discrimination of their VOC profiles. *E. coli* strains emitted the greatest diversity of VOCs, with long chain alcohols, ketones, and indole being the most characteristic VOCs recovered. *S. epidermidis* emitted a relatively low number of VOCs and had the least discriminative VOC profile. Strain-level variation in VOC profiles was limited, observed differences in the VOC profiles of *E. coli* and *P. aeruginosa* strains suggest potential specificity in certain bacterial strains. Although the examined strains of *E. coli* and *P. aeruginosa* highlight potential measurable strain-level differences in the emission of specific VOCs, the number of strains analysed in our study is not sufficient to draw conclusions. As expected, our results demonstrate that bacterial VOC profiles are highly discriminative at the species-level and relatively less discriminative at the strain-level. Future investigations of strain-level diversity of VOC profiles must incorporate a higher number of strains and account for genomic similarities between examined strains. Profiling the emission of certain compound classes by *S. aureus* and *P. aeruginosa* over time demonstrated a proportional relationship between the emission of particular compound classes and the respective growth phase of the cells. The results obtained using this robust HS-SPME GCMS workflow are comprehensive with high numbers of identified compounds being recovered, giving high levels of discriminatory power to the method, highlighting its strong potential application for future untargeted microbial studies. The next step in our research is to carry out an in vivo untargeted VOC profiling study with the aim of characterising VOC profiles from patient DFU swab samples of varying severities and microbial compositions.

## Methods

### Growth of bacteria

The following bacterial strains were studied: *S. aureus (*DSM2569 and DSM799)*; P. aeruginosa* (DSM19880 and DSM25642)*; E. coli* (DSM30083 and DSM105372); and *S. epidermidis* (CSF41498 and RP62A)*.* All *S. aureus, P. aeruginosa, and E. coli* isolates were obtained from Leibniz Institute DSMZ-German Collection of Microorganisms and Cell Cultures GmbH; *S. epidermidis* strains were provided by Prof. O’Gara at NUI Galway. Each strain was streaked individually on tryptone soy broth (TSB) agar media plates and incubated at 37 °C overnight. Overnight liquid cultures were prepared in 4 mL of TSB broth and incubated at 37 °C overnight with shaking (180 rpm). The samples are referred throughout the text using the following acronyms*: EC.A: E. coli DSM103372, EC.B: E. coli DSM30083, PA.A: P. aeruginosa DSM105372, PA.B: P. aeruginosa DSM25642, SA.A: S. aureus DSM2569, SA.B: S. aureus DSM799, SEP.A: S. epidermidis, CSF41498, SEP.B: S. epidermidis RP62A and TSB:* growth media control. Samples for VOC analysis were prepared in 20 mL sterile headspace (HS) vials (Merck, Cork, Ireland). Overnight cultures were diluted in 5 mL of TSB media to a final cell count of approximately 10^7^–10^10^ colony forming units (CFU)/mL in the HS vials which were then sealed with magnetic polytetrafluoroethylene/silicone septum screw caps (Merck, Cork, Ireland). Samples were set up in triplicate and incubated at 37 °C with shaking for a set period of time. Nine black media controls were also sampled and analysed at these conditions. Growth curve analysis was performed using *S. aureus* and *P. aeruginosa.* Samples were set up in triplicate and incubated at 37 °C with shaking. A spectrometer is used to measure the optical density of a given culture at 600 nm (OD_600_). OD_600_ was taken at 2, 4, 6, 8, 24, 32, 48 h.

### Sampling procedure

SPME fibers were used for sampling VOCs and comprised of 85 μm Carboxen/PDMS Stableflex (2 cm) assemblies (Supelco Corp., Bellefonte, PA, USA). Prior to sampling, each bacterial or control sample was removed from the shaking incubator and placed in a standard incubator at 37 °C. The SPME needle was pierced through the septum of the HS vial, and the fibre was exposed to the HS of the sample for 20 min, after which, the fibre was retracted and the SPME assembly removed from the vial. The SPME fibre was then inserted into the GC inlet and thermally desorbed at 250 °C for 2 min for subsequent separation and detection by mass spectrometry.

### Gas chromatography–mass spectrometry

An Agilent 6890 GC connected to an Agilent 5973 mass selective detector (Agilent Technologies, Inc., Santa Clara, CA, USA) was used for all analyses. Separations were performed on a DB-WAX column (Agilent Technologies Ireland, Cork) (30 m × 0.25 mm × 0.32 μm). The carrier gas used was helium, with a constant flow rate of 1.3 mL/min For manual injections of SPME fibers, the system was equipped with a SPME Merlin Microseal (Merlin Instrument Company, Newark, DE, USA), and the inlet was maintained at a temperature of 250 °C. Split-less injection was used for all samples, with a gas purge being activated after 2 min. Each SPME fibre was desorbed for 2 min within a SPME inlet liner (Supelco). The initial GC oven temperature was 40 °C for 5 min and was programmed to increase at a rate of 10 °C min^−1^ to 240 °C, with a final hold for 5 min at this temperature, giving an overall running time of 29 min. The MS was operated at a scan range of 35–400 m*/z*, scan rate of 3.94 s^−1^, ion source temperature 230 °C and ionising energy of 70 eV. Identification of compounds was performed using the National Institute of Standards and Technology (NIST) library (2017)—match factors of > 70% were used. Retention index (RI) matching was used to support the identification of these compounds. Any compound found to have an RI value ≤ 12 RI units of the RI values found in the NIST database were deemed acceptable matches. A standard mixture of saturated alkanes (C_7_–C_30_; Merck, Cork, Ireland) was used for RI matching.

### Data analysis

The open source software OpenChrom^66^ was used to analyse raw chromatographic data. Chromatographic peaks were compared using the NIST Chemistry WebBook. Peaks found to be from exogenous sources such as the SPME fiber, glass vial, and column were removed from the dataset. RStudio was used for data exploration and visualisation. Raw bacterial VOC data was standardised using centering and scaling^67^. Centering converts all the values in the dataset to fluctuations around zero rather than fluctuations around the mean VOC abundance. It adjusts for differences in the offset between low and high abundances. Scaling converts the values in the dataset into ratios relative to the difference in abundances between the VOCs, which allows each VOC to be equally represented in the subsequent data analysis. Hierarchical clustering and principal component analysis (PCA) were carried out on the dataset using the R packages: ‘FactoMineR’, ‘factoextra’, ‘pheatmap’, ‘egg’ and ‘cluster’. Other R packages used included: ‘tidyverse’, ‘ggplot2’, ‘ggfortify’.

## Supplementary information

Supplementary Information.
