# Supplementary material for: Multi-strain volatile profiling of pathogenic and commensal cutaneous bacteria
Source: Sci Rep. 2020 Oct 21;10:17971. doi: 10.1038/s41598-020-74909-w (PMC7578783; doi:10.1038/s41598-020-74909-w)
Supplement: Supplementary file 1 — Supplementary Information. [file 41598_2020_74909_MOESM1_ESM.docx]

Multi -strain volatile profiling of pathogenic and commensal cutaneous bacteria

# Shane Fitzgerald^[1]^, Emer Duffy^[1]^, Linda Holland^[2]^, Aoife Morrin*^[1]^

## School of Chemical Sciences, National Centre for Sensor Research, Insight SFI Research Centre for Data Analytics, Dublin City University, Ireland

## School of Biotechnology, Dublin City University, Ireland

*aoife.morrin@dcu.ie

**Supplementary Information**

**

*Figure S1: Overlaid chromatograms of SA.A (blue, top) and SA.B (red, bottom)*

**

*Figure S2: Overlaid chromatograms of PA.A (blue, top) and PA.B (red, bottom)*

**

*Figure S3: Overlaid chromatograms of EC.A (blue, top) and EC.B (red, bottom)*

**

*Figure S4: Overlaid chromatograms of SEP.A (blue, top) and SEP.B (red, bottom)*

**

*Figure S5: Heatmap showing the relative abundance of VOCs recovered (rows) from each bacterial strain (columns). Values were scaled and centred by their respective rows, with highly abundant VOCs being coloured red, and less abundant VOCs being marked orange - yellow .*

**

*Figure S6: Loadings plot of bacteria only samples. Bacteria-specific VOCs are indicated with blue lines and are distributed across the plot with respect to their presence in specific species and the relative abundance emitted. Compounds detected in media control samples have been subtracted.*

**

*Figure S7: Kinetic plots for individual VOC emissions from S. aureus samples (n=3) over 48 h.*

**

*Figure S8: Kinetic plots for individual VOC emissions from P. aeruginosa samples (n=3) over 48 h.*

Table S1: Percentage normalised peak area values of each compound identified in the HS of liquid bacterial cultures (following 24 h incubation at 37°C) after 20 min sample collection using the HS-SPME technique followed by thermal desorption to GC-MS. Compounds are listed in order of increasing retention time. Kovats retention index (RI) value range (polar column) for each compound is also provided.

| **Compound** | **CAS** | **RI** | **SA.A** | **SA.B** | **PA.A** | **PA.B** | **EC.A** | **EC.B** | **SEP.A** | **SEP.B** | **TSB** |
| --- | --- | --- | --- | --- | --- | --- | --- | --- | --- | --- | --- |
| 2-Butanone | 78-93-3 | 900 ± 20 | 1.642 | 1.262 | 0.000 | 0.000 | 1.629 | 0.204 | 1.496 | 0.000 | 1.314 |
| 3-Methylbutyraldehyde | 590-86-3 | 920 ± 20 | 3.229 | 2.635 | 0.000 | 0.000 | 0.000 | 0.000 | 1.789 | 2.580 | 18.521 |
| 2-Butanone, 3-methyl- | 563-80-4 | 950 ± 20 | 2.910 | 1.419 | 0.000 | 0.000 | 0.000 | 1.875 | 0.000 | 2.013 | 1.221 |
| Decane | 124-18-5 | - | 0.000 | 0.000 | 0.000 | 1.725 | 0.000 | 0.000 | 1.056 | 2.533 | 0.000 |
| Methyl Isobutyl Ketone | 108-10-1 | 1010 ± 15 | 0.680 | 0.000 | 0.000 | 0.683 | 0.000 | 0.309 | 0.000 | 0.455 | 0.000 |
| 2-Butanol | 78-92-2 | 1026 ± 15 | 0.000 | 0.000 | 0.167 | 0.263 | 0.000 | 0.000 | 0.000 | 0.000 | 0.000 |
| Butanoic acid, 2-methyl-, ethyl ester | 7452-79-1 | 1050 ± 25 | 0.444 | 0.701 | 0.000 | 0.000 | 0.000 | 0.000 | 0.383 | 0.291 | 0.000 |
| Disulfide, dimethyl | 624-92-0 | 1060 ± 30 | 1.655 | 2.170 | 1.261 | 0.766 | 3.210 | 0.705 | 4.897 | 1.036 | 4.505 |
| 1-Propanol, 2-methyl- | 78-83-1 | 1090 ± 30 | 0.534 | 0.952 | 0.000 | 0.000 | 0.000 | 0.000 | 0.000 | 0.000 | 0.000 |
| 3-Penten-2-one, 4-methyl- | 141-79-7 | 1125 ± 25 | 0.230 | 0.000 | 0.000 | 0.720 | 0.000 | 0.343 | 0.000 | 0.703 | 0.000 |
| 2-pentanol | 6032-29-7 | 1130 ± 25 | 0.000 | 0.000 | 0.000 | 0.000 | 0.000 | 0.015 | 0.000 | 0.459 | 0.000 |
| 1-Butanol | 71-36-3 | 1125 ± 25 | 0.000 | 0.000 | 0.000 | 0.000 | 1.432 | 0.546 | 0.000 | 0.225 | 0.000 |
| 1-Butanol, 3-methyl-, acetate | 821-95-4 | 1120 ± 15 | 0.000 | 0.179 | 0.000 | 0.000 | 0.000 | 0.000 | 0.000 | 0.000 | 0.000 |
| 1-Undecene | 112-40-3 | 1142 ± 12 | 0.000 | 0.000 | 3.975 | 14.926 | 0.000 | 0.000 | 0.000 | 0.000 | 0.000 |
| Dodecane | 110-43-0 | - | 0.000 | 0.000 | 0.000 | 0.000 | 0.000 | 0.000 | 0.410 | 0.000 | 0.000 |
| 2-Heptanone | 6137-06-0 | 1184 ± 30 | 0.258 | 0.049 | 0.000 | 0.000 | 0.542 | 0.177 | 0.208 | 0.423 | 0.000 |
| 2-Heptanone, 4-methyl- | 18217-12-4 | 1213 ± 10 | 2.354 | 0.281 | 0.131 | 2.436 | 0.000 | 1.497 | 0.000 | 3.292 | 0.074 |
| 2-Heptanone, 5-methyl- | 290-37-9 | 1252 | 0.000 | 0.000 | 0.000 | 0.000 | 0.000 | 0.000 | 0.000 | 0.308 | 0.000 |
| Pyrazine | 123-51-3 | 1214 ± 22 | 0.664 | 0.320 | 1.095 | 0.000 | 0.000 | 0.000 | 0.000 | 0.000 | 1.887 |
| 1-Butanol, 3-methyl- | 100-42-5 | 1199 ± 20 | 4.720 | 11.476 | 12.873 | 6.688 | 6.265 | 1.119 | 1.066 | 0.291 | 0.000 |
| Styrene | 763-32-6 | 1265 ± 35 | 0.000 | 0.000 | 0.000 | 0.000 | 0.341 | 0.000 | 0.000 | 0.000 | 0.105 |
| 3-Buten-1-ol, 3-methyl- | 109-08-0 | 1250 ± 25 | 0.426 | 0.460 | 0.000 | 0.225 | 0.000 | 0.077 | 0.435 | 0.207 | 0.150 |
| Pyrazine, methyl- | 116-09-6 | 1260 ± 25 | 1.472 | 1.130 | 1.578 | 0.693 | 0.766 | 0.398 | 1.228 | 0.796 | 1.462 |
| 2-Propanone, 1-hydroxy- | 513-86-0 | 1290 ± 25 | 0.073 | 0.105 | 0.000 | 0.000 | 0.157 | 0.157 | 0.264 | 0.240 | 0.322 |
| 2-Butanone, 3-hydroxy- | 629-50-5 | 1290 ± 15 | 1.598 | 1.661 | 0.000 | 0.000 | 0.097 | 0.095 | 0.000 | 0.229 | 0.000 |
| Tridecane | 123-32-0 | - | 0.000 | 0.000 | 0.279 | 0.000 | 0.000 | 0.000 | 0.000 | 0.000 | 0.000 |
| Pyrazine, 2,5-dimethyl- | 111-27-3 | 1320 ± 20 | 12.279 | 11.466 | 15.562 | 2.393 | 4.865 | 3.188 | 10.909 | 3.771 | 13.510 |
| 1-Hexanol | 3658-80-8 | 1350 ± 20 | 0.000 | 0.000 | 0.000 | 0.000 | 0.157 | 0.058 | 0.000 | 0.000 | 0.000 |
| Dimethyl trisulfide | 821-55-6 | 1365 ± 25 | 0.175 | 0.000 | 0.043 | 0.000 | 0.378 | 0.000 | 0.000 | 0.000 | 0.142 |
| 2-Nonanone | 629-59-4 | 1380 ± 20 | 0.000 | 0.000 | 0.224 | 0.000 | 1.593 | 0.000 | 0.000 | 0.000 | 0.000 |
| Tetradecane | 5704-20-1 | - | 0.000 | 0.000 | 0.000 | 0.000 | 0.079 | 0.020 | 0.000 | 0.000 | 0.000 |
| 2-Hydroxy-3-pentanone | 13925-03-6 | 1380 ± 20 | 0.000 | 0.164 | 0.000 | 0.000 | 0.000 | 0.000 | 0.000 | 0.000 | 0.000 |
| Pyrazine, 2-ethyl-6-methyl- | 13360-64-0 | 1385 ± 15 | 0.011 | 0.109 | 0.000 | 0.000 | 0.000 | 0.000 | 0.371 | 0.000 | 0.124 |
| Pyrazine, 2-ethyl-5-methyl- | 14667-55-1 | 1390 ± 15 | 0.641 | 0.318 | 0.237 | 0.000 | 0.000 | 0.000 | 0.000 | 0.000 | 0.285 |
| Pyrazine, trimethyl- | 106-32-1 | 1400 ± 20 | 0.621 | 0.616 | 0.632 | 0.181 | 0.174 | 0.108 | 0.233 | 0.202 | 0.604 |
| Octanoic acid, ethyl ester | 628-99-9 | 1425 ± 20 | 0.000 | 0.000 | 0.000 | 0.000 | 0.101 | 0.054 | 0.000 | 0.000 | 0.000 |
| Pyrazine, 3-ethyl-2,5-dimethyl- | 64-19-7 | 1455 ± 25 | 0.177 | 0.595 | 0.265 | 0.000 | 0.000 | 0.087 | 0.000 | 0.000 | 0.676 |
| Acetic acid | 112-31-2 | 1460 ± 30 | 3.687 | 1.481 | 0.000 | 0.000 | 2.703 | 0.470 | 5.811 | 6.569 | 0.000 |
| Decanal | 100-52-7 | 1470 ± 20 | 0.000 | 0.000 | 0.000 | 0.000 | 0.126 | 0.036 | 0.000 | 0.000 | 0.000 |
| 2-Decanone | 79-31-2 | 1490 ± 25 | 0.000 | 0.000 | 0.000 | 0.000 | 1.322 | 0.248 | 0.000 | 0.000 | 0.000 |
| 2-Nonanol | 13360-65-1 | 1505 ± 20 | 0.000 | 0.000 | 0.196 | 0.368 | 0.315 | 0.000 | 0.000 | 0.000 | 0.000 |
| Pyrrole | 93-58-3 | 1515 ± 25 | 0.000 | 0.000 | 0.279 | 0.415 | 0.000 | 0.000 | 0.000 | 0.000 | 0.000 |
| Benzaldehyde | 629-62-9 | 1520 ± 25 | 0.000 | 0.000 | 0.000 | 0.000 | 0.000 | 0.446 | 0.000 | 0.000 | 15.211 |
| Pentadecane | 111-87-5 | - | 0.000 | 0.000 | 0.421 | 0.000 | 0.000 | 0.000 | 0.000 | 0.000 | 0.000 |
| 1-Octanol | 109-97-7 | 1550 ± 25 | 0.000 | 0.000 | 0.000 | 0.000 | 1.390 | 0.380 | 0.000 | 0.000 | 0.000 |
| Benzoic acid, methyl ester | 693-54-9 | 1615 ± 25 | 0.000 | 0.000 | 0.000 | 0.000 | 0.000 | 0.000 | 0.412 | 0.000 | 0.000 |
| Propanoic acid, 2-methyl- | 636-41-9 | 1560 ± 25 | 0.201 | 0.375 | 0.000 | 0.000 | 0.000 | 0.000 | 0.143 | 0.301 | 0.000 |
| 1H-Pyrrole, 2-methyl- | 112-12-9 | 1555 ± 15 | 0.000 | 0.000 | 0.205 | 0.000 | 0.000 | 0.000 | 0.000 | 0.000 | 0.000 |
| 2-Undecanone | 503-74-2 | 1590 ± 15 | 0.000 | 0.000 | 0.043 | 0.192 | 0.380 | 0.213 | 0.000 | 0.000 | 0.000 |
| 3-Methylbutyric acid | 1653-30-1 | 1660 ± 25 | 2.460 | 7.043 | 0.000 | 0.000 | 0.000 | 0.000 | 4.277 | 7.039 | 0.000 |
| 2-Undecanol | 112-30-1 | 1712 ± 15 | 0.000 | 0.000 | 0.000 | 0.241 | 0.562 | 0.067 | 0.000 | 0.000 | 0.000 |
| 1-Decanol | 111-82-0 | 1750 ± 20 | 0.000 | 0.000 | 0.000 | 0.000 | 6.722 | 0.000 | 0.000 | 0.000 | 0.000 |
| Dodecanoic acid, methyl ester | 593-08-8 | 1800 ± 15 | 0.000 | 0.000 | 0.000 | 0.000 | 0.264 | 0.010 | 0.000 | 0.000 | 0.000 |
| 2-Tridecanone | 2345-27-9 | 1805 ± 15 | 0.000 | 0.000 | 0.000 | 0.000 | 1.497 | 0.316 | 0.000 | 0.000 | 0.000 |
| Benzyl Alcohol | 100-51-6 | 1850 ± 30 | 0.132 | 0.000 | 0.000 | 0.000 | 0.144 | 0.138 | 0.000 | 0.000 | 0.000 |
| Phenylethyl Alcohol | 60-12-8 | 1900 ± 25 | 0.000 | 0.000 | 0.000 | 0.000 | 0.236 | 0.113 | 0.000 | 0.000 | 0.000 |
| 2-Tridecanol | 1653-31-2 | 1904 ± 10 | 0.000 | 0.000 | 0.000 | 0.000 | 0.501 | 0.000 | 0.000 | 0.000 | 0.000 |
| 1-Dodecanol | 112-53-8 | 1965 ± 20 | 0.000 | 0.000 | 0.000 | 0.000 | 3.642 | 1.159 | 0.000 | 0.000 | 0.057 |
| Methyl tetradecanoate | 124-10-7 | 2010 ± 25 | 0.000 | 0.000 | 0.000 | 0.000 | 1.496 | 0.014 | 0.000 | 0.000 | 0.000 |
| 2-Pentadecanone | 2345-28-0 | 2020 ± 20 | 0.000 | 0.000 | 0.000 | 0.000 | 0.423 | 0.023 | 0.000 | 0.000 | 0.000 |
| Phenol | 108-95-2 | 2000 ± 30 | 0.042 | 0.060 | 0.060 | 0.000 | 0.000 | 0.000 | 0.000 | 0.000 | 0.055 |
| 1-Tetradecanol | 112-72-1 | 2175 ± 30 | 0.000 | 0.000 | 0.000 | 0.000 | 0.275 | 0.513 | 0.000 | 0.000 | 0.000 |
| Hexadecanoic acid, methyl ester | 112-39-0 | 2250 ± 45 | 0.000 | 0.000 | 0.000 | 0.000 | 0.683 | 0.000 | 0.000 | 0.000 | 0.000 |
| 1-Hexadecanol | 36653-82-4 | 2375 ± 25 | 0.000 | 0.000 | 0.000 | 0.000 | 0.000 | 0.460 | 0.000 | 0.000 | 0.000 |
| Indole | 120-72-9 | 2420 ± 25 | 0.000 | 0.000 | 0.000 | 0.000 | 88.617 | 41.254 | 0.000 | 0.000 | 0.000 |
